# A comprehensive quantitative bottom-up analysis of fiber-reinforced recycled-aggregate concrete behavior

**DOI:** 10.1038/s41598-023-31646-0

**Published:** 2023-03-18

**Authors:** Maedeh Hosseinzadeh, Mehdi Dehestani, Hojjat Samadvand

**Affiliations:** 1 Faculty of Civil Engineering, Babol Noshirvani University of Technology, Postal Box: 484, Babol, 47148-71167 Iran; 2 Department of Civil Engineering, EFC College, Urmia, Iran

**Keywords:** Engineering, Civil engineering, Composites, Mechanical properties

## Abstract

This study provides a more profound understanding of the influence of the phases of fiber-reinforced recycled-aggregate concrete (FRRAC), on its elastic properties, in particular Young’s modulus and Poisson’s ratio. Multi-scale modeling analyses of mortar and FRRAC were conducted to assess the effect of variations in the fiber content, fiber elastic modulus, RA content, and water-to-cement ratio (w/c) on the elastic properties at each scale. Thus, the analytic Mori–Tanaka (MT) homogenization algorithm developed in Python programming language and the three-dimensional finite element (FE) homogenization scheme were applied to evaluate the elastic properties of FRRAC. As such, different fiber types including steel, basalt, glass, and propylene, at a volume fraction range of 0–2%, along with the variations in fiber elastic modulus, and different RA replacement levels ranging from 0 to 100% were incorporated in the modeling process at different w/c ratio. Based on the results, the Poisson’s ratio of steel FRRAC in the MT approach surges with increasing fiber content. Furthermore, the elastic modulus of FRRAC is highly susceptible to an increase in Young’s modulus of polypropylene fiber, among other fiber types. The elastic modulus of concrete experiences a sharp decrease with increasing w/c for all fiber types in both FE and MT approaches.

## Introduction

Construction and demolition wastes, as one of the largest shares of municipal solid wastes throughout the world, pose a serious threat to the global environment^1,2^. Given that disposing of concrete demolition wastes is difficult, and also the persistent utilization of natural aggregate resources is non-sustainable, it is necessary to recycle such wastes within the construction industry^3^. Reusing these wastes to produce fresh concrete is known as an attractive approach in terms of environment and economy^4,5^. Thus, the demand for recycling construction and demolition wastes in the form of recycled aggregates (RA) is high^6^. In this respect, recycled aggregate concrete (RAC) is typically a type of concrete containing aggregates obtained from crushing and processing of previously used concrete structural elements that partially or completely replace conventional natural aggregates (NA)^7^. In the meantime, utilizing RA reduces waste, leading to more protection of the environment and more sustainable practices in the field of building construction. Not only can this eliminate billions of tons of construction and demolition wastes produced globally^2^, but also CO_2_ emission and limestone consumption can be reduced by up to 20% and 60%, respectively^7,8^.

The properties of RA are different from those of virgin aggregates, mainly because of the existing old mortar^9^. In general, concrete specifications depend on the properties of aggregates used in the mix design^3^. By the same token, RA properties significantly affect the mechanical and chemical properties of concrete. Studies have exhibited that the mechanical strength of RAC is lower than that of virgin aggregates^10^, primarily due to the adhered old cement mortar^11,12^. The lower performance of RAC, as opposed to conventional concrete, is a major issue in recycling construction and demolition waste in the production of RAC products. For example, the inferior properties of RA result in a 30–40% reduction in the compressive strength of RAC compared to natural aggregate concrete (NAC)^13^. In this regard, the application of RA had been dismayed until 2013, as implicated in BS EN 206:2013^14^, so the engineers preferred NAC over RAC. However, with all the requisites of sustainability, RAC is being endorsed in recent years and guidelines are being developed for its application in structural concrete by numerous standards^7,15^. Furthermore, the researchers have already adopted effective techniques to improve the RAC properties involving the addition of fibers into RAC^16–19^. For example, Zheng et al.^19^ evaluated the mechanical response of basalt fiber-reinforced recycled aggregate concrete (BFRRAC) at three micro, meso, and macro scales. They stated that fiber volume fraction with an optimal content of 0.2% was the main factor influencing the mechanical properties of BFRRAC. Xie et al.^20^ performed a qualitative and quantitative data review on the effect of incorporating fibers on the mechanical strength and durability properties of RAC. They also reported several performance evaluation models regarding fiber-reinforced recycled aggregate concrete (FRRAC). It was concluded that the crack-bridging mechanism provided by fibers, particularly steel fibers, helps improve strength and durability. However, basalt fibers are more sustainable. Ahmed and Lim^21^ comprehensively studied the mechanical properties of FRRAC, highlighting the most practical strength improvement techniques using various types of fibers, including steel, polypropylene (PP), basalt, and glass fibers. They also presented several relationships between fiber volume fractions, aggregate replacement levels, and strength enhancement in RAC to obtain the optimal fiber dosage for the strength enhancement in FRRAC. Zhang et al.^22^ conducted an in-depth analysis of the mechanical characteristics of basalt fiber-reinforced recycled concrete (BFRRC). Their results showed that the major mechanical properties of RAC containing 50% recycled aggregate replacement are better than those of the NAC at different fiber addition volumes. Yongggui et al.^23^ proposed a novel method of improving the RAC properties using fibers and nanoparticles. The maximum compressive BFRRAC strength was achieved when the RA replacement ratio was 50%. Also, the fiber content and RA replacement ratio were determined as the main factors influencing the splitting tensile and flexural strengths of BFRRAC. Zhang et al.^24^ used the reaction surface technique to effectively predict the mechanical strength of hybrid basalt-steel FRRAC. A three-dimensional (3D) prediction model of the FRRAC mechanical strength was developed using a multiple nonlinear regression method, and the variations in fiber addition were examined. Xu et al.^25,26^ applied multiple nonlinear regression and artificial neural networks frameworks in estimating the mechanical properties of RAC. Xu et al.^27^ presented unified models to predict the compressive behavior of confined and unconfined RAC. Thilakarathna et al.^28^ investigated the elastic modulus and Poisson’s ratio of NAC based on FE modeling and homogenization theory. Subsequently, the predictions of the random distribution meso-model of fiber-reinforced concrete were compared with the macroscopic experimental values. Similarly, Li and Li^29^ evaluated the elasticity properties of ultra high strength concrete with the aid of finite element (FE) representative volume element (RVE) analysis to predict the volume fractions of different phases in generating RVE geometries to be employed in FE-RVE simulations. A five-level multi-scale framework was then proposed using experiments and simulation results as inputs in the framework. A subsequent parametric analysis was also performed to examine the effect of boundary conditions and the shape of inhomogeneities within RVEs on the homogenized elastic modulus. Chen et al.^30^ experimented with the mechanical strength of BFRRC. It was revealed that basalt fibers have a better reinforcement effect on the early strength of RAC than NAC. They also stated that the combined incorporation of basalt fibers at low dosages and RA in high contents leads to a specific strength higher than that of RAC. Sun et al.^31,32^ developed a homogenization algorithm based on Mori–Tanaka (MT) and a multi-scale FE simulation approach to estimate the impact of different specimen sizes and fiber volume fractions on the fracture performance of BFFRC. Alternative aggregate and fiber types have also been utilized in producing RAC. In this sense, Ahmed et al.^33^ utilized different proportions of PP fiber in high-strength RAC mixes. It was shown that adding PP fibers increased the compressive strength of the RAC by 20.8%, 15.2%, and 11.6% when recycled aggregate replacement was 0%, 50%, and 100%, respectively. Li et al.^34^ evaluated the Young’s modulus of concrete containing silica fume via multi-scale MT and self-consistent (SC) homogenization schemes considering the thickness of the interfacial transition zone (ITZ) around coarse aggregates. The results indicated that using silica fume increased the Young’s moduli of the C–S–H gel and ITZ but led to the reduction in ITZ thickness. Chen et al.^35^ evaluated the effect of coarse recycled concrete aggregate on the compressive strength of C20/25–C80/95 concrete classes. The aggregate volume fraction was recommended as a realistic quantification index in concrete mixtures. Xiao^3^ stated that the water-cement ratio (w/c) of RAC needs to be lower than that of NAC so as to achieve similar strength capacity. Xiong et al.^36^ indicated that the strain-rate susceptibility of RAC is higher than that of the NAC. Silva et al.^37^ established a relation for the elastic modulus based on the compressive strength. Wang et al.^38^ aimed to estimate the elastic modulus of concrete containing coal gangue aggregates at different replacement ratios. The 28-day elastic modulus of concrete was decreased by 23–32% based on the proposed two-phase composite model. Luo et al.^39^ investigated the effect of aggregate shape and diameter and interfacial defects on the mesoscale concrete properties with homogeneous elastic modulus. The results demonstrated that a reduction in aggregate diameter improved the concrete strength, and the aggregate-mortar interfacial defect reduced the tensile strength by 67.9%. Gote et al.^40^ carried out a numerical homogenization analysis of concrete. They confirmed that key parameters in the computational homogenization of concrete include phase fractions, homogenized elasticity tensor, tensor invariance relative to boundary conditions, and the total/spatial error distributions.

### Research significance

The use of machine learning and numerical modeling techniques to develop different engineering models of FRRAC has recently gained much importance^41–44^. In this research, a 3D modeling of FRRAC and its constituent phases at meso and macro scales was performed, with the purpose to capture the specifications of actual models through analytic and numerical simulations. Furthermore, the effect of variations in fiber content, fiber elastic modulus, RA content, and w/c ratio on the elastic properties of FRRAC was investigated. The strategy taken for a detailed examination of the aggregate size range was to divide the NA and RA size intervals into several groups, based on the available PSD curve data. Then, the center of each group was chosen as the aggregate diameter. Hence, the percentage retained in each sieve between the two consecutive sizes was considered as that group percentage to be included in the modeling procedure. The proposed methodology can supplement or replace the current costly experimental modeling procedures, which require stringent laboratory equipment to conduct experiments at the mesoscale. In addition, a multi-scale modeling approach was adopted to evaluate RA properties in a distinct and more accurate procedure. The use of a multi-scale modeling procedure moderated intricacy, and escalated the accuracy of the outcome compared to the conventional modeling approaches. Also, valuable information on better understanding the RA characteristics and the effect of the constituent phases on the elasticity of FRRAC was provided. Afterward, the FRRAC homogenization was investigated using the analytic Mori–Tanaka (MT) homogenization algorithm developed in Python programming language. Finally, a 3D numerical modeling was performed with the help of FEM to estimate the elastic constants.

## Research methodology

### Mori–Tanaka (MT) homogenization approach

**Figure 1:**
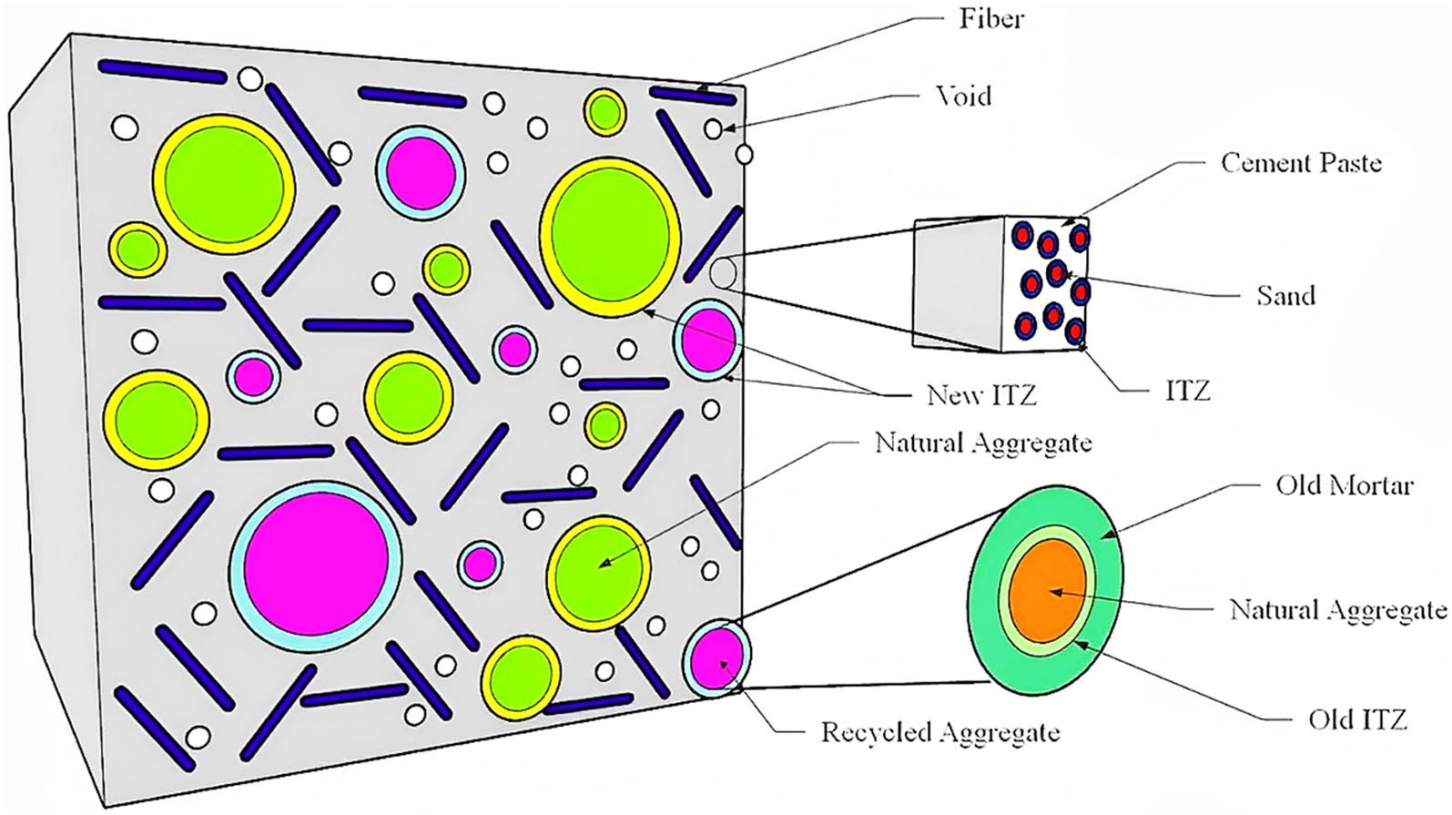
Schematic of FRRAC model.

The Mori–Tanaka (MT) method coded in Python was applied to investigate the elastic properties of the RAC model in terms of Young’s modulus, volume fraction, and Poisson’s ratio of each constituent phase. Thus, a 3D model was implemented to examine the elastic properties. In this regard, using the known mechanical specifications of sand, ITZ, and cement paste at the mesoscale, the properties of mortar were estimated to be used at the macroscale. Accordingly, mortar, which includes cement paste, sand, and the respective ITZ, was first homogenized in mesoscale using FEM relations. Then, the elastic properties of the homogenized material obtained in this step were implemented for modeling at macroscale. The FRRAC model in macroscale was developed pertinent to w/c ratio and fine aggregate volume, so that summation of the inclusions and matrix volume came to unity. The schematic of FRRAC model is presented in Fig. 1. Also, Table 1 reports the elastic properties of all constituent phases at macroscale.

**Table 1:** Elastic properties of constituent phases at macroscale.

Phases	Elastic modulus (GPa)	Poisson’s ratio (–)
NA		
Agg	70	0.16
New ITZ	17.97	0.23
RA		
Agg	70	0.16
Old ITZ	24.77	0.2198
Old mortar		
New ITZ		
New mortar	35.93	0.253
Voids	0	0

The MT algorithm uses input parameters shown in Table 2. It is to note that the cement paste and the homogenized mortar were regarded as matrices at meso and macro levels, respectively. Also, the rest of the phases were considered inclusions in the modeling process of the FRRAC.

**Table 2:** Input data used for MT algorithm and FE scheme.

W/C	Cement paste		ITZ			Sand		
	E (Gpa)	ν	E (Gpa)	ν	Size (m)	E (Gpa)	ν	Size (m)
0.35	30.3879	0.27	15.194	0.245	0.000026	86.7	0.17	0.00085
0.4	26.3796	0.27	13.19	0.245	0.000026	86.7	0.17	0.00085
0.45	22.8662	0.27	11.433	0.245	0.000026	86.7	0.17	0.00085
0.5	19.7652	0.27	9.883	0.245	0.000026	86.7	0.17	0.00085
0.55	17.1178	0.27	8.559	0.245	0.000026	86.7	0.17	0.00085
0.6	14.924	0.27	7.462	0.245	0.000026	86.7	0.17	0.00085

In addition to the values in Table 2, the Eshelby tensors of all inclusions are required in this approach^45^. In this sense, the Eshelby tensors of voids, aggregates, old mortar, and their relative ITZs were assumed spheres. However, a cylindrical Eshelby tensor was employed to model the fibers. Hence, the stiffness matrices of the comprising phases and their combination were examined. Finally, the stiffness matrix of the FRRAC was estimated in the MT approach. The stiffness matrix in the MT approach can be described as Eq. (1), as follows:

1 $$ \overline{\mathbb{C}} = \left( {\sum_{\alpha = 0}^{n} \;f_{\alpha } {\mathbb{C}}^{\alpha } :{\mathcal{A}}^{\alpha } } \right):\left( {\sum_{\alpha = 0}^{n} \;f_{\alpha } {\mathcal{A}}^{\alpha } } \right)^{ - 1} $$

in which (C) denotes the stiffness tensor in terms of concentration tensor (**A**^I^), given in Eq. (2):

2 $$ {\mathcal{A}}^{I} = \left[ {{\mathbb{I}}^{{\left( {4s} \right)}} + {\mathbb{P}}^{I} :\left( {{\mathbb{C}}^{I} - {\mathbb{C}}} \right)} \right]^{ - 1} $$

where (P^I^) corresponds to the polarization tensor, defined by Eq. (3):

3 $$ {\mathbb{P}}^{I} = {\mathbb{S}}^{I,\infty } :{\mathbb{C}}^{ - 1} $$

It is to note that *I* represents the identity tensor. Additionally, the components of the spherical Eshelby tensor (*S*) contained in the polarization tensor are determined by Eqs. (4)–(6).

4 $$ S_{1111} = S_{2222} = S_{3333} = \frac{7 - 5v}{{15\left( {1 - v} \right)}} $$

5 $$ S_{1122} = S_{2233} = S_{3311} = S_{1133} = S_{2211} = S_{3322} = \frac{5v - 1}{{15\left( {1 - v} \right)}} $$

6 $$ S_{1212} = S_{2323} = S_{3131} = \frac{4 - 5v}{{15\left( {1 - v} \right)}} $$

Likewise, the cylindrical Eshelby tensor (${S}^{^{\prime}}$) components are represented in Eqs. (7)–(16).

7 $$ S_{{1111 }} = \frac{1}{{2\left( {1 - \nu } \right)}}\left[ {\frac{{b^{2} + 2ab}}{{(a + b)^{2} }} + \left( {1 - 2\nu } \right)\frac{b}{a + b}} \right] $$

8 $$ S_{1212} = \frac{1}{{2\left( {1 - \nu } \right)}}\left[ {\frac{{a^{2} + b^{2} }}{{2(a + b)^{2} }} + \frac{{\left( {1 - 2\nu } \right)}}{2}} \right] $$

9 $$ S_{{1133 }} = \frac{1}{{2\left( {1 - \nu } \right)}}\frac{2\nu b}{{a + b}} $$

10 $$ S_{2323} = \frac{a}{{2\left( {a + b} \right)}} $$

11 $$ S_{3131} = \frac{b}{{2\left( {a + b} \right)}} $$

12 $$ S_{{2222 }} = \frac{1}{{2\left( {1 - \nu } \right)}}\left[ {\frac{{a^{2} + 2ab}}{{(a + b)^{2} }} + \left( {1 - 2\nu } \right)\frac{a}{a + b}} \right] $$

13 $$ S_{{3333 = }} S_{{3311 = }} S_{{3322 = 0 }} $$

14 $$ S_{{1122 }} = \frac{1}{{2\left( {1 - \nu } \right)}}\left[ {\frac{{b^{2} }}{{(a + b)^{2} }} + \left( {1 - 2\nu } \right)\frac{b}{a + b}} \right] $$

15 $$ S_{2233} = \frac{1}{{2\left( {1 - \nu } \right)}}\frac{{2_{\nu a} }}{a + b} $$

16 $$ S_{{2211 }} = \frac{1}{{2\left( {1 - \nu } \right)}}\left[ {\frac{{a^{2} }}{{(a + b)^{2} }} + \left( {1 - 2\nu } \right)\frac{a}{a + b}} \right] $$

**Figure 2:**
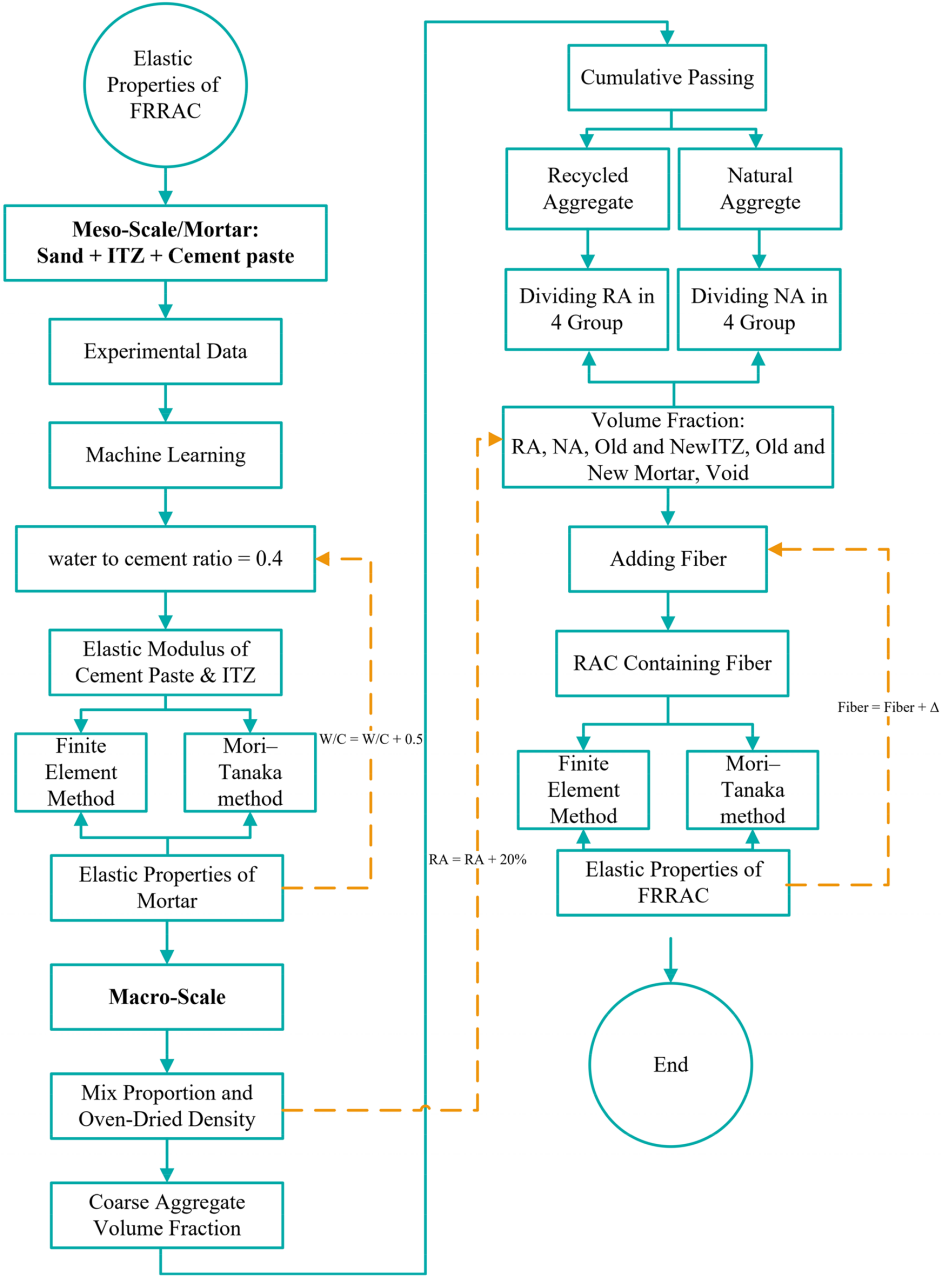
The overall flowchart of the proposed multi-scale modeling approach.

The C^α^ tensor changes each time the elastic moduli of the constituent FRRAC phases vary at every single input point of the algorithm. Consequently, the A^α^ tensor also changes. Thus, new values are reproduced as output operation points to be used in the next step of the algorithm. The overall flow diagram of the multi-scale modeling approach proposed in this study is demonstrated in Fig. 2.

### Finite element (FE) modeling approach

In this research, the elastic properties of mortar and FRRAC were estimated using FE and MT homogenization approaches. The multi-scale material modeling DigiMat-FE software (version 2016) was implemented at each scale to create the RVEs. The data used at each scale can be obtained using the previous studies and experimental results^46^. A high-performance computing (HPC) cluster was employed in the modeling procedure. Also, none of the inclusions intersect each other, so the periodic geometry was used in generating RVEs. It should also be noted that the spherical inclusion shape was employed in the modeling of aggregates and voids, and the sphero-cylinder shape was used for the modeling of fibers, whose time and cost consumption are lower than the other shapes. The meshing of the generated RVEs was handled using a regular, non-conforming voxel mesh so as to avert badly-shaped elements. RVE sizes of 2.5, 5, and 10 mm were defined at the mesoscale. The meshing effect on each phase volume fraction in the RVE was determined by comparison of the respective volume fractions. By some means, this criterion is indicative of the quality of mesh discretization. Once the convergence analysis was carried out for the different meshing densities, the final RVE size of 10 mm was considered for the mesoscale. Likewise, RVE sizes of 25, 50, and 100 mm were modeled at the macroscale, and the optimum convergent size of 100 mm was selected. Subsequently, each one of the examined RVE sizes was divided by 50, 75, and 100 in order to obtain the mesh size. The elastic properties of the phases were then investigated, the results of which are reported in Table 3.

**Table 3:** Elastic properties based on RVE and mesh size at mesoscale.

RVE size (mm)	Mesh size (mm)	Elastic modulus (GPa)	Poisson’s ratio (–)
2.5	0.05	39.1	0.25
	0.03	38.8	0.25
	0.03	38.5	0.26
5	0.1	39.7	0.25
	0.07	39.3	0.26
	0.05	39.1	0.26
10	0.2	40.5	0.25
	0.13	39.7	0.26
	0.1	39.6	0.26

The mechanical type of analysis was adopted to apply the mechanical load and to compute the mechanical response of the generated 3D RVEs. Also, cohesive material was used to model the inclusion-matrix and fiber-matrix debonding. Thereby, the inter-phase was replaced by a layer of cohesive elements COH3D6. In addition, the inter-phase, which is itself influenced by the presence of inclusions, was defined with a cohesive behavior with an absolute thickness of [3 + (2 × 5 × 10^−3^)] = 3.1 mm]. Debonding at interface was also included. The random 3D orientation was selected for fibers in the modeling space. For the mechanical analysis type, proper loading and boundary conditions (BCs) should be specified and used in the FE model. The loading type in Digimat was a macroscopic strain state along the *z* axis imposed to the RVE.

#### Mesoscale FE modeling

An RVE was created at mesoscale using the FE modeling approach. The RVE comprising cement paste, sand, and ITZ, was then developed to examine the effects of mortar elastic modulus, sand content, and w/c ratio. Experimental results in the literature were used as input to characterize the effect of cement paste and w/c ratio on the elastic properties. However, due to lack of w/c ratios in the literature for all points, machine learning (ML) and Python programming were applied to estimate different w/c ratios. Therefore, mortar was modeled and homogenized with different dimensions based on data available from the previous experiments. Given that mortar consists of three main parts of cement paste, sand, and ITZ, the characteristics of each of these phases were obtained by previous studies, and their effect on mortar and RAC were investigated in this research. Finally, the model created via FE modeling was validated with the results obtained from the experimental studies in the technical literature.

#### Macroscale FE modeling

Similarly, a macroscale RVE model was created including mortar, RA, NA, void, fiber, and the relative ITZs. The model was then extended to capture the effects of RA replacement level, and w/c ratio through FE approach. Also, sand content was explored in MT approach at mesoscale. Subsequently, RAC was modeled and homogenized with different dimensions based on data available from the previous experiments. Considering that RAC is comprised of seven main phases of mortar, RA, NA, relative ITZs, fiber, and voids, the effects of RA content, fiber content, fiber elastic properties, and w/c ratio on RAC were explored in this study. Finally, the RAC model created via FE modeling was verified with the results obtained from the experimental studies in the literature.

### Modeling of cement paste

#### Effect of w/c on elastic properties of cement paste via ML methodology

**Figure 3:**
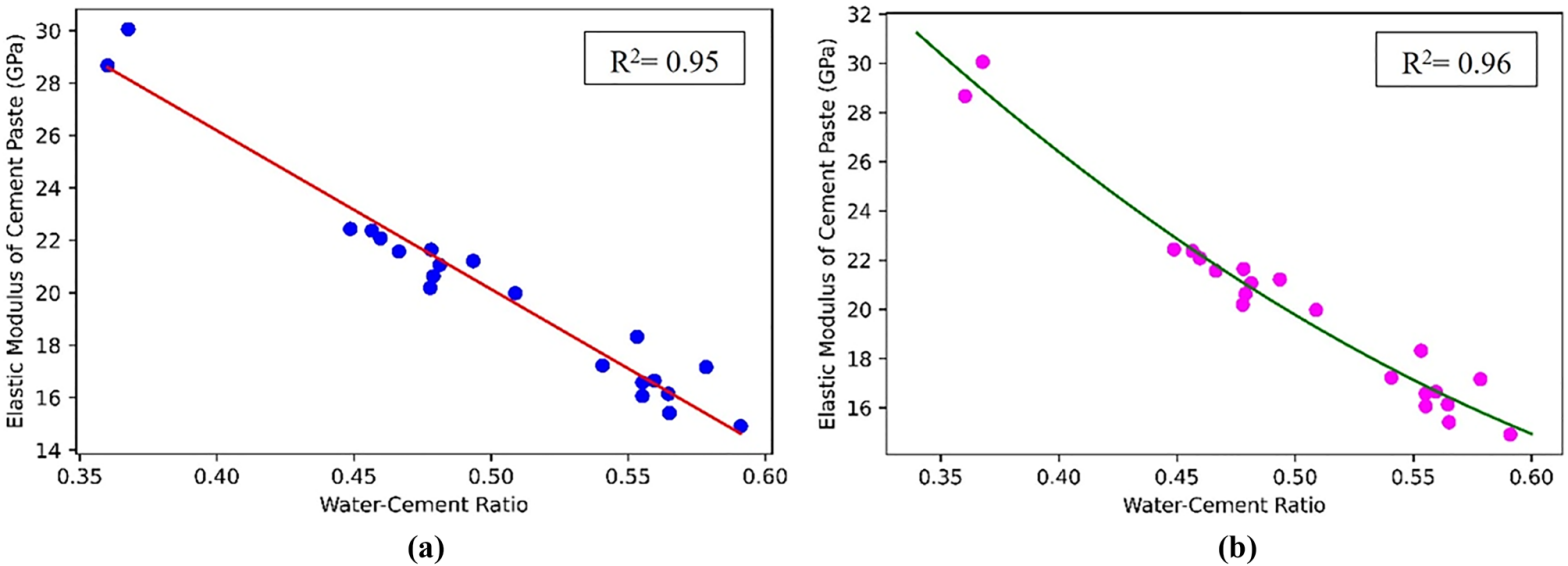
Prediction curves between w/c ratio and the elastic modulus of cement paste in (**a**) linear regression; (**b**) polynomial regression.

Using test results in the literature^47^ and ML coupled with simple linear and more complex polynomial regression analyses, the relationship between the w/c ratio and elastic modulus of cement paste was estimated. In the linear regression approach, the generic optimization algorithm, capable of finding optimal solutions to a wide range of problems, was implemented to train the model and minimize the cost function over the training set. In like manner, polynomial regression fits the training data much better than linear regression. Once the model was trained, the unregularized performance measure was used to evaluate the model performance. In addition, the training was stopped as soon as the validation error reached the questioned minimum. Python programming and the experimental data obtained from previous studies^47–49^ were also used to perform an efficient error analysis. In this wise, 20% of the data was used for testing and 80% was used for the training set. Data regression was carried out by using the Python programming library. Then, using this method, the coefficients for the linear and polynomial regressions were computed. The amount of error was also examined for each case. As shown in Fig. 3, the prediction curve between the w/c ratio and the elastic modulus of cement paste in polynomial regression is relatively a better model than the linear regression at predicting the output. Accordingly, the R-squared value for linear and polynomial regression models was 0.95 and 0.96, respectively, demonstrating the higher accuracy of the polynomial model. Hence, the polynomial method was used in this research. Figure 3a,b illustrate the relationship between the w/c ratio and the elastic modulus of cement paste.

Once the relationship between the w/c ratio and the elastic modulus of cement paste was obtained, the elastic modulus and Poisson’s ratio of mortar were calculated for all w/c ratios of 0.3, 0.35, 0.4, 0.45, 0.5, 0.55, and 0.6 to be used in the modeling process, given by Table 4.

**Table 4:** Comparing MT and FE results for the effect of w/c ratio on the elastic properties at mesoscale.

W/C	Current study			
	FEM		MT	
	Mortar elastic modulus (GPa)	Mortar Poisson’s ratio (–)	Mortar elastic modulus (GPa)	Mortar Poisson’s ratio (–)
0.35	39.8901	0.253403	38.89298633	0.246978123
0.4	35.9341	0.253403	34.75049069	0.247330161
0.45	32.6374	0.253403	30.95017603	0.247896793
0.5	29.3407	0.253403	27.43851813	0.248506668
0.55	26.3736	0.253403	24.31888701	0.248962872
0.6	24.0659	0.253403	21.631071	0.249346292

#### Effect of w/c on elastic properties of cement paste via MT algorithm

Experimental data of previous studies were used to investigate the effect of the w/c ratio on the elastic modulus of cement paste through the MT homogenization algorithm and FE approach. Thus, the elastic properties of mortar and its constituent phases, including shear and bulk moduli, were obtained from experiments carried out by Hashin and Monteiro^46^, as expressed in Eqs. (17) and (18), and reported in Table 5. Therefore, by using shear and bulk moduli, the elastic properties of sand, cement paste, ITZ, and mortar were predicted.

17 $$ E_{{\text{mor }}} = \frac{{9K_{{\text{mor }}} G_{{\text{mor }}} }}{{3K_{{\text{mor }}} + G_{mor} }} $$

18 $$ \begin{array}{*{20}c} \\ {\nu_{{\text{mor }}} = \frac{{3K_{{\text{mor }}} - 2G_{{\text{mor }}} }}{{2\left( {3K_{mor} + G_{mor} } \right)}}} \\ \end{array} $$

where $K_{{\text{mor }}}$ and $G_{{\text{mor }}}$ represent bulk and shear moduli of mortar, respectively.

**Table 5:** Comparison of the experimental and measured elastic properties of mortar at different sand volume fractions.

Volume fraction (%)	Current study (MT)		Hashin and Monteiro^46^			
	Mortar elastic modulus (GPa)	Mortar Poisson’s ratio (–)	Mortar Bulk modulus (GPa)	Mortar shear modulus (GPa)	Mortar elastic modulus (GPa)	Mortar Poisson’s ratio (–)
15	30.73463955	0.257538429	24.14	13.35	33.816	0.267
27	34.75049069	0.247330161	26.81	14.87	37.649	0.266
40	39.76774036	0.235814295	27.69	16.91	42.15	0.246
52	45.16782898	0.224532877	29.96	19.26	47.584	0.235
65	52.08383667	0.211276287	30.12	20.23	49.588	0.226

According to the results obtained by^46^, the elastic modulus of ITZ is approximately half that of the cement mortar. Considering the Poisson's ratio of ITZ, this measure varies from 0.2 to 0.25 in different studies. Also, it is known that the Poisson's ratio of mortar is by 0.2/0.22 higher than that of ITZ^50^. Therefore, given that the Poisson’s ratio of mortar is 0.27 in this study, the Poisson's ratio of 0.245 was adopted for ITZ. Accordingly, the elastic modulus and Poisson's ratio of mortar were estimated against different w/c ratios and volume fractions of sand, using the equations in MT approach and the relations of spherical Eshelby tensor in Python, assuming sand and ITZ as the inclusions and cement paste as the matrix. The elastic properties of mortar were also obtained by FE approach in terms of different w/c ratios. The results are elaborated on in “Results and discussion” section.

#### Effect of w/c on elastic properties of cement paste via FE approach

**Figure 4:**
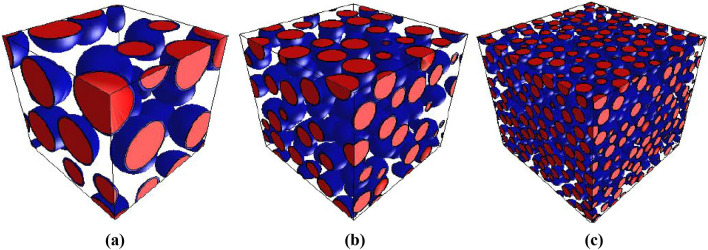
RVE sizes of mortar at mesoscale: (**a**) 2.5, (**b**) 5, and (**c**) 10 mm.

**Figure 5:**
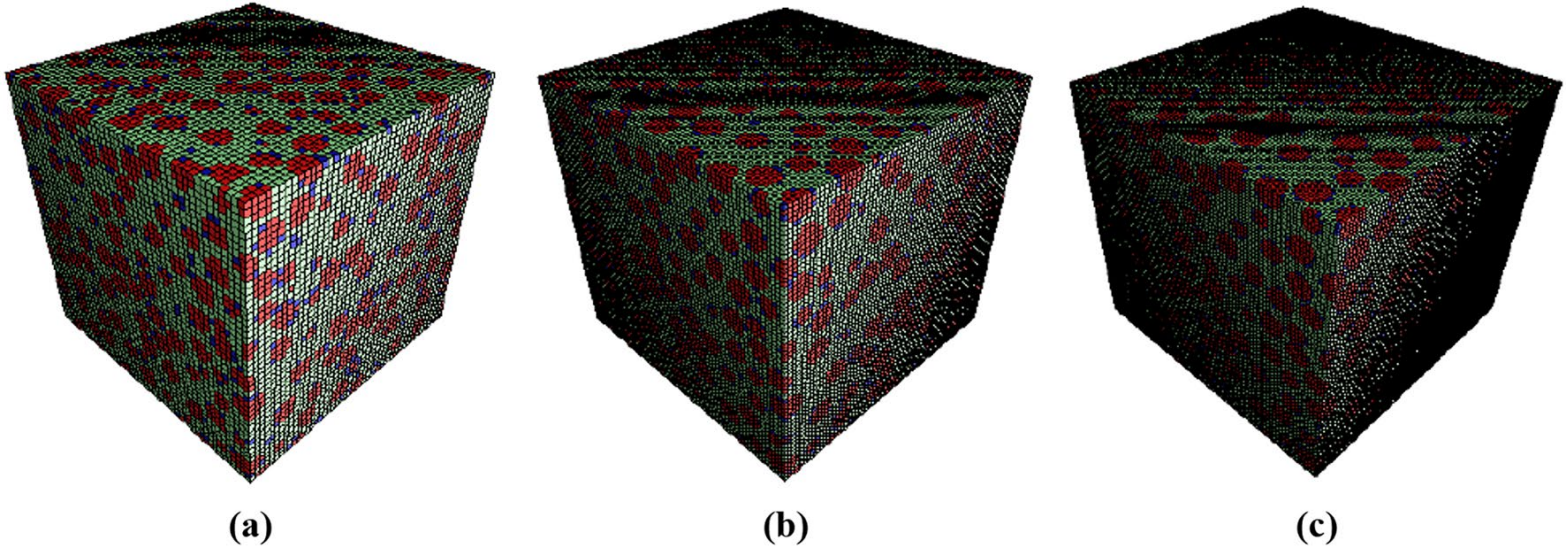
Mesh sizes at mesoscale: (**a**) 0.2, (**b**) 0.133, and (**c**) 0.1 mm.

Using the FE method, RVE models of mortar consisting of sand, cement paste, and ITZ were created and meshed in different sizes, as shown in Figs. 4 and 5. The model with dimensions 10 mm and mesh size 0.133 mm was adopted as the mesoscale RVE. In addition, the effect of the w/c ratio on the corresponding elastic properties was compared for the two MT and FE approaches, as given in Table 4.

#### Effect of fine aggregate on elastic properties of mortar

In addition to the effect of w/c ratio, the aggregate content can play a significant role in modifying the elastic properties of mortar. Based on the literature, fine aggregate volume can range between 0.15 and 0.62. In this study, the effect of aggregate content on the elastic properties of mortar was investigated via MT algorithm.

### Modeling of FRRAC

#### MT modeling approach

After examining the influencing factors, mortar was modeled on a mesoscale. The results obtained on this scale were used as input data on a macroscale, including eight phases of NA, RA, new ITZ, old ITZ, new mortar, old mortar, voids, and fiber. In this scale, the RAC reinforced with four types of steel, basalt, glass, and PP fibers was modeled and validated using experimental data from the previous studies. The physical and mechanical properties of fibers are reported in Table 6. FRRAC is a heterogeneous material that comprises all the mentioned phases. In this research, all phases were modeled with a 3D FE approach and then homogenized with the MT algorithm and Python programming language.

**Table 6:** Physical and mechanical properties of fibers.

Fiber type	Steel	Basalt	Glass	PP
Elastic modulus (GPa)	210	85	72	10
Poisson’s ratio (–)	0.3	0.26	0.21	0.2
Aspect ratio	45–80	21–69	42–85	28–67
Length (mm)	32–60	18–32	6–18	12–50
Reference	^43–59^	^23,60,61^	^33,62–69^	^70,71^

The elastic properties of the constituent phases of FRRAC are reported in Table 1, based on the experiments carried out so far. Mix design proposed by Chen et al.^35^ was followed in this study to determine the volume fraction of each phase in FRRAC. Given that the weight of each constituent is known in the mix design, volume fractions of NA and RA were determined using the oven-dried density expressed in Table 7.

**Table 7:** RA and NA size and oven-dried density.

Aggregate type	Aggregate density (kg/m^3^)	Aggregate size (mm)
RA	2380	6.3–14
NA	2630	6.3–14

**Figure 6:**
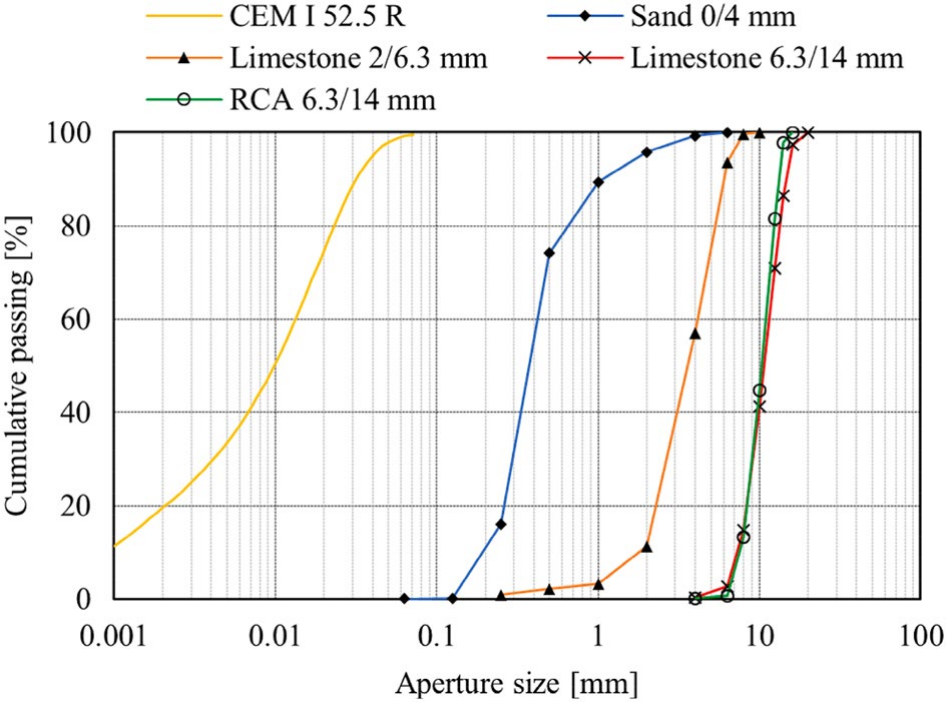
Particle size distribution curve^35^.

Moreover, the aggregate size range varies from 6.3 to 14 mm, based on the particle size distribution (PSD) curve illustrated in Fig. 6. For the sake of a detailed examination of the aggregate size range, each of the NA and RA size ranges was divided into four groups, using the PSD curve data in Fig. 6, so as to create a model as close to the real one. Then, the center of each group interval was chosen as the aggregate size (diameter), according to Table 8. In addition, the percent retained in each sieve between the two aggregate sizes was considered as that group percentage, and included in the modeling. Given that RAC with different RA contents has been examined in this research, as reported in Table 9, each group percentage was specified based on the RA replacement level for either of the RA and NA categories. Since all sizes incorporate in the particle distribution of NA, a single layer, as the new ITZ, is thus added to the aggregate in the modeling process of FRRAC. However, given that RA consists of three phases of NA, old ITZ, and old mortar (a portion of the aggregate diameter is made up of old ITZ and old mortar), an additional layer is also modeled.

**Table 8:** Determination of RA and NA size percentage at each group.

Data obtained from PSD curve		Center of each group		RA (%)					
Size (mm)	Cumulative passing (%)	Size-diameter (mm)	Group percent	0%	20%	40%	60%	80%	100%
RA									
6.3	0	7.15	15.13	0	0.88	1.76	2.63	3.51	4.39
8	15.13	9	33.37	0	1.94	3.87	5.81	7.74	9.68
10	48.5	11	27.33	0	1.59	3.17	4.76	6.34	7.93
12	75.83	13	24.17	0	1.4	2.8	4.21	5.61	7.01
14	100	–	100	0	5.8	11.6	17.4	23.2	29
∑RA (%)				0	0.058	0.174	0.116	0.232	0.29

Data obtained from PSD curve		Center of each group		NA (%)					
Size (mm)	Cumulative passing (%)	Size-diameter (mm)	Group percent	100%	80%	60%	40%	20%	0%
NA									
6.3	0	7.15	16.05	4.6545	3.72	2.79	1.86	0.93	0
8	16.05	9	24.17	7.0093	5.61	4.21	2.8	1.4	0
10	40.22	11	26.2	7.598	6.08	4.56	3.04	1.52	0
12	66.42	13	33.58	9.7382	7.79	5.84	3.9	1.95	0
14	100	–	100	29	23.2	17.4	11.6	5.8	0
RA + NA				0.29	0.29	0.29	0.29	0.29	0.29

**Table 9:** RA and NA volumetric content, Chen et al.^35^.

RA content (%)	NA content (%)	RA mass (kg)	NA mass (kg)	RA volume (%)	NA volume (%)	Total coarse aggregate (%)
0	100	0	763	0.000	0.290	0.29
20	80	138	610	0.058	0.232	0.29
40	60	276	458	0.116	0.174	0.29
60	40	415	305	0.174	0.116	0.29
80	20	553	153	0.232	0.058	0.29
100	0	691	0	0.290	0.000	0.29

Table 9 shows the volumetric content of each of the RA and NA, obtained from the concrete mix design proposed by Chen et al.^35^.

Based on the studies available in the literature, the thickness of the old mortar can range between 3 and 7 mm relative to coarse aggregate size. Since the coarse aggregate used in this research lies in the range of 6.3–14 mm, a thickness of 3 mm was selected for old mortar. According to Chen et al.^35^, the thickness of the old and new ITZs was taken as 50 μm, by subtracting of which from the RA diameter, the NA percentage embedded in RA can be achieved.

As acknowledged in the literature, the effect of the ITZ on the elastic modulus and Poisson's ratio is negligible^42^. Therefore, an equivalent layer with homogenized elastic properties and a thickness equal to the total thicknesses of old aggregate, old ITZ, and new ITZ phases was modeled through the MT algorithm, as expressed in Table 10.

**Table 10:** Homogenization of the RA constituent layers.

Phases	Elastic Modulus (GPa)	Poisson’s ratio (–)	Thickness (m)
Old ITZ	17.5	0.2	5.00E−05
Old mortar	25	0.22	3.00E−03
New ITZ	17.96705	0.23	5.00E−05
Homogenized layer	24.766	0.2198	3.10E−03

#### FE modeling approach

**Figure 7:**
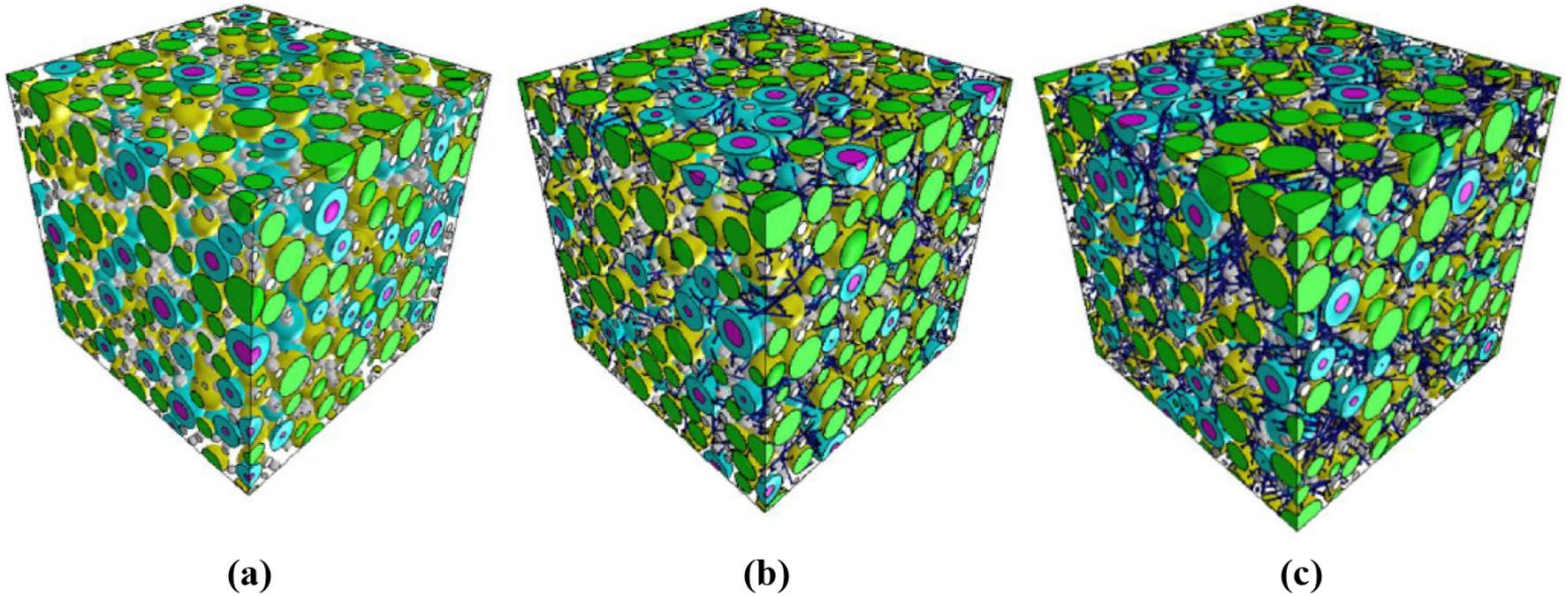
FRRAC at fiber contents of: (**a**) 0, (**b**) 1, and (**c**) 2%.

**Figure 8:**
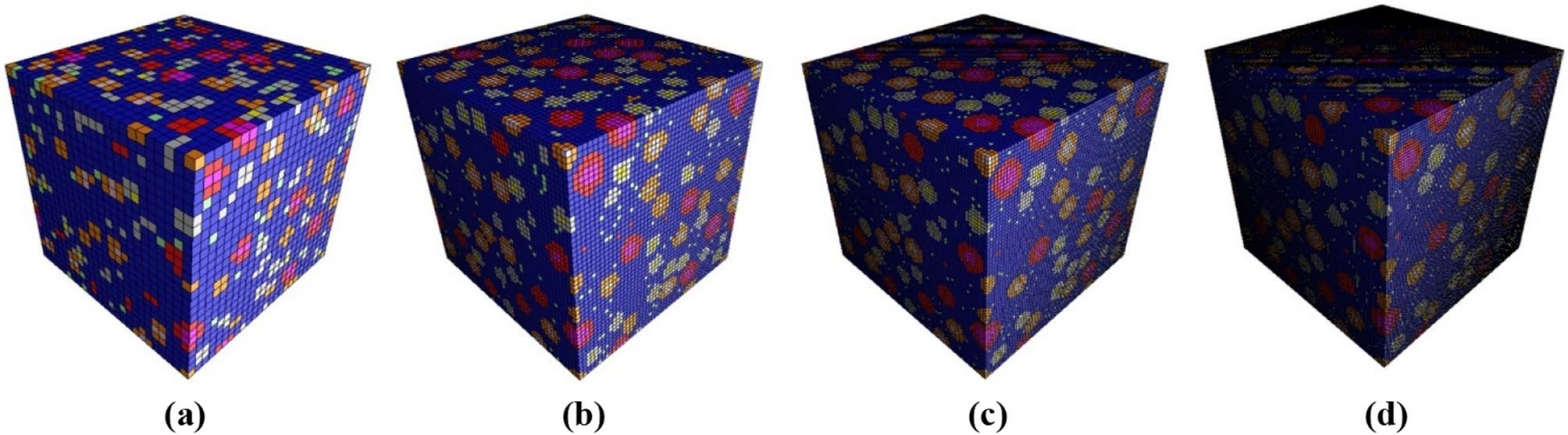
FRRAC RVE models at macroscale at different sizes of: (**a**) 4, (**b**) 2, (**c**) 1.33 and (**d**) 1 mm.

Using the FE method, RVE models of FRRAC consisting of the cited eight phases (that is, NA, RA, new ITZ, old ITZ, new mortar, old mortar, voids, and fiber) were created in different sizes, as shown in Fig. 7. The model with dimensions 100 mm and mesh size 1.33 mm was adopted as the macroscale RVE. The effects of w/c ratio, fiber content, RA content, and fiber elastic modulus on the elastic properties of mortar and FRRAC were examined, the results of which are presented in “Results and discussion” section. Besides, Fig. 8 illustrates the RVE model of FRRAC at the macroscale.

## Verification

### Mesoscale modeling verification

Using Eqs. (17) and (18), as also the shear and bulk moduli of each phase, the elastic properties of each phase were calculated. A model characterizing the obtained properties and the available experimental data was created, and the modeling results were compared to those of the literature in Table 11. According to Table 11, the elastic modulus of the mortar was obtained 38.125 GPa at mesoscale, while the analogous value experimented by^46^ is 37.649 GPa. This shows a good agreement between the proposed approach and the experimental results with a negligible discrepancy of 1%.

**Table 11:** Validation of the results at mesoscale between the current study and the experiment^46^.

Phase properties	Input data			Output data	
	Sand	ITZ	Cement paste	Mortar-experiment^46^	Mortar-current study
Elastic modulus (GPa)	86.698	1.388	30.134	37.649	38.125
Poisson’s ratio (–)	0.1716	0.394	0.2769	0.2659	–
Bulk modulus (GPa)	44	–	22.51	26.81	–
Shear modulus (GPa)	37	0.498	11.8	14.87	–
Volume fraction (%)	0.27	–	–	–	–
Size (m)	8.50E−04	2.60E−05	–	–	–

### Macroscale modeling verification

Based on the proposed homogenization approach, extreme values for the elastic modulus of RAC were calculated and compared with the experiments conducted by Chen et al.^35^. Thus, a minimum of 15.8 GPa and a maximum of 35.9 GPa were proposed in that study for the Young’s modulus of RAC. Then, the elastic modulus of the RAC expressed in terms of the compressive strength proposed by Silva et al.^37^ was computed in this study as 34.074 GPa for a given compressive strength of 80 MPa, following the Eq. (19). It is seen that the experimental value lies within the range proposed in the current research. The elastic properties of the RAC phases, along with the variation interval for the elastic modulus, are reported in Table 12.

19 $$ {\text{E}}_{{{\text{cm}}}} = 0.830 \times 22\left( {{\text{f}}_{{{\text{cm}}}} /10} \right)^{ \wedge } 0.3 $$

in which ${\mathrm{E}}_{\mathrm{cm}}$ and ${\mathrm{f}}_{\mathrm{cm}}$ denote the Young’s modulus and the compressive strength of RAC.

**Table 12:** Elastic properties of RAC phases along with variation interval for the elastic modulus.

Min			Max		
Phases	Elastic modulus (GPa)^35^	Elastic modulus (GPa) Current study	Phases	Elastic modulus (GPa)^35^	Elastic modulus (GPa) Current study
Aggregate	40	16.5	Aggregate	100	38
Mortar + ITZs	17.35		Mortar + ITZs	24.84	
New mortar	16.1		New mortar	36.8	
Voids	1.00E−05	–	Voids	1.00E−05	–
Variation range^35^	15.8		Variation range^35^	35.9	

As for another example of the validation process, the analytical MT homogenization method and the FE approach were implemented to compare the results obtained in this study with those of the multi-scale framework presented by Thilakarathna et al.^28^. The thri-phasic mesoscale RVE comprising coarse aggregates, mortar matrix, and voids was modeled accordingly. Also, perfect bonding of aggregate and matrix was adopted for simplicity, while accounting for the interactions between inhomogeneities within the generated RVE. The RVE size was taken as 50 mm and the coarse aggregate diameter ranged from 7 to 12 mm. The pore size of 2–5 mm was also considered. Table 13 summarizes the elastic properties at mesoscale based on Thilakarathna et al.^28^. According to the elastic modulus results obtained in Table 14, the python programming homogenization exercised in this study is by 3% different than that of the analytical MT homogenization reported by Thilakarathna et al.^28^. Also, the elastic modulus of the RAC obtained in this study via Digimat is 6% higher than that estimated by the FE approach in the literature.

**Table 13:** RAC properties at mesoscale, Thilakarathna et al.^28^.

Phase	Elastic modulus (GPa)	Poisson’s ratio (–)	Volume fraction (%)	Size (mm)
Aggregate	100.8	0.25	0.4	7–12
Mortar + ITZs	–	–	0	–
New mortar	42.9	0.23	0.5	–
Voids	0	0	0.1	2–5

**Table 14:** Validation of the elastic property results in the current study and Thilakarathna et al.^28^.

Approach	Mori–Tanaka		FE	
	Elastic modulus homogenization (GPa)	Poisson’s ratio homogenization (–)	Elastic modulus homogenization (GPa)	Poisson’s ratio homogenization (–)
Thilakarathna et al.^28^	47.1	0.23	48.3	0.22
Current study	45.604	0.285	51.2211	–

For further validation of the proposed methodology, the elastic properties of FRRAC containing 1 and 2% steel fibers were compared with the homogenization theory and experimental values of the elastic modulus and Poisson’s ratio examined by Li and Li^29^. In this model, the elastic moduli of 210 and 31.1 GPa were considered for steel fibers and concrete, respectively. Also, the Poisson’s ratio of fibers and concrete was assumed 0.3 and 0.2, respectively. According to Table 15, the elastic modulus of FRRAC containing 1% fiber was 31.52 and 31.68 GPa for the python programming algorithm and the FE-Digimat modeling performed in this study. These values show an excellent agreement with the experimental measures of Thomas and Ramaswamy^72^ and the analytical homogenization results with a maximum of 1% discrepancy. Likewise, the elastic modulus of the FRRAC containing 2% fiber was obtained 31.94 and 32.18 GPa for the python programming and FE-Digimat modeling, respectively. The corresponding experimental^72^ and analytical values^29^ were 32.1 and 33.02 GPa, demonstrating a maximum difference of 3% from those attained in this research.

**Table 15:** Elastic modulus comparison between current study, experiment^72^, and homogenization approach^29^.

Elastic properties of phases				SFRC			
				MT approach (current study)	FE digimat (current study)	Experiment^72^	Homogenization^29^
Phase	Elastic modulus (GPa)	Poisson’s ratio (–)	Fiber content (%)	Elastic modulus (GPa)			
Steel fiber	210	0.3	1	31.52	31.68	31.5	31.95
Concrete	31.1	0.2	2	31.94	32.18	32.1	33.02

The validation of the framework presented in this study was once more confirmed by the results of elastic modulus and Poisson’s ratio of an FRRAC with two different 28-day compressive strengths of 35 and 65 MPa containing steel fibers at volume fractions of 0.5, 1, and 1.5%, as reported in Table 16. The elastic modulus and Poisson’s ratio of the RVE structures modeled in this example were 28.7 GPa and 0.182 for the 35-MPa concrete, and 37.5 MPa and 0.201 for the 65-MPa concrete. Based on the results reported in Table 16, the present framework can generate admissible results in terms of the elastic specifications of the FRRAC.

**Table 16:** Validation of elastic modulus and Poisson’s ratio results for FRRAC.

Elastic properties of phases				SFRC							
				MT		Experiment^72^				Homogenization^29^	
Phase	E (GPa)	ν (–)	Fiber content (%)	E(GPa)	ν (–)	E(GPa)	ν (–)	E-error (%)	ν-error (%)	E (GPa)	ν (–)
Steel fiber	210	0.3	–								
Concrete (1)	31.1	0.2	1	31.52	0.202	31.5	0.2	− 0.1	0.8	31.95	0.21
			2	31.94	0.204	32.1	0.21	0.5	5.8	33.02	0.22
Concrete (35 MPa)	28.7	0.182	0.5	28.89	0.18	29.4	0.186	1.7	1.5	28.85	0.187
			1	29.09	0.18	30.2	0.19	3.7	3	29.65	0.196
			1.5	29.28	0.18	31.1	0.195	5.8	5	30.2	0.203
Concrete (65 MPa)	37.5	0.201	0.5	37.73	0.202	38.6	0.204	2.2	1	37.92	0.206
			1	37.97	0.203	39.8	0.213	4.6	4.8	38.43	0.213
			1.5	38.2	0.204	41	0.219	6.8	6.9	38.95	0.219

## Results and discussion

This section presents the results of FRRAC elastic properties, including Young’s modulus and Poisson’s ratio. Thus, 3D multi-scale modeling output were compared using the two MT Python programming and FE homogenization approaches in terms of variations in the fiber type, content, and elastic modulus, as well as the different RA replacement levels and w/c ratios.

### The effect of fiber content

**Figure 9:**
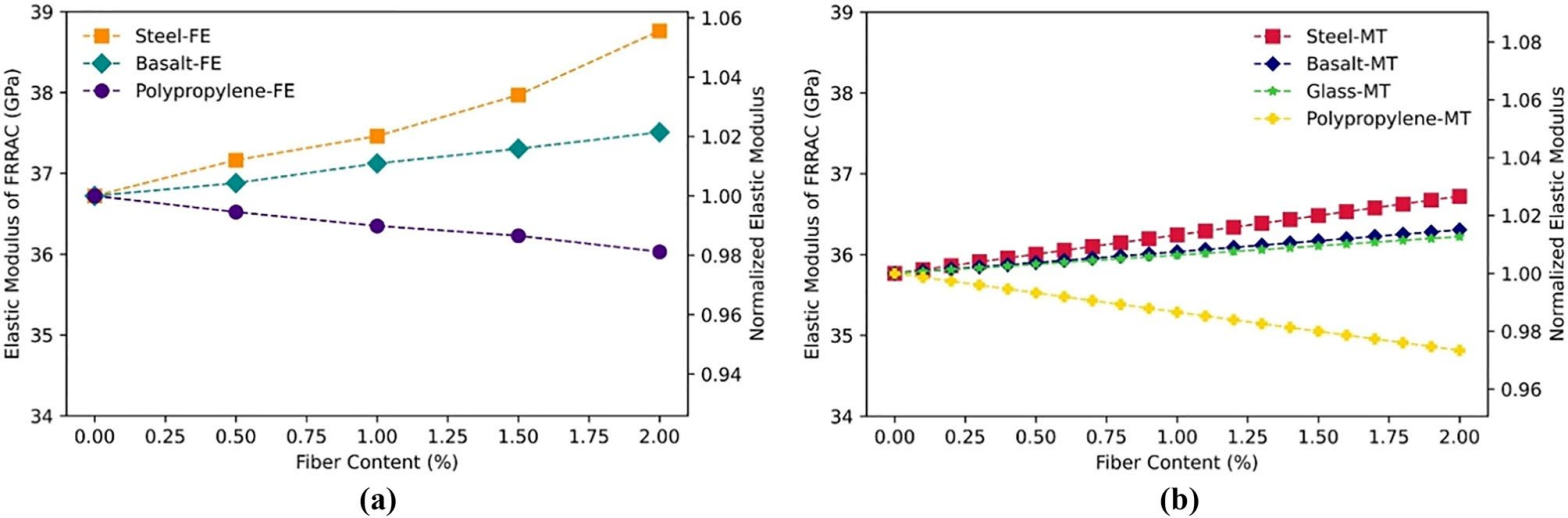
The elastic modulus of FRRAC based on fiber content: (**a**) FE approach, (**b**) MT approach.

**Figure 10:**
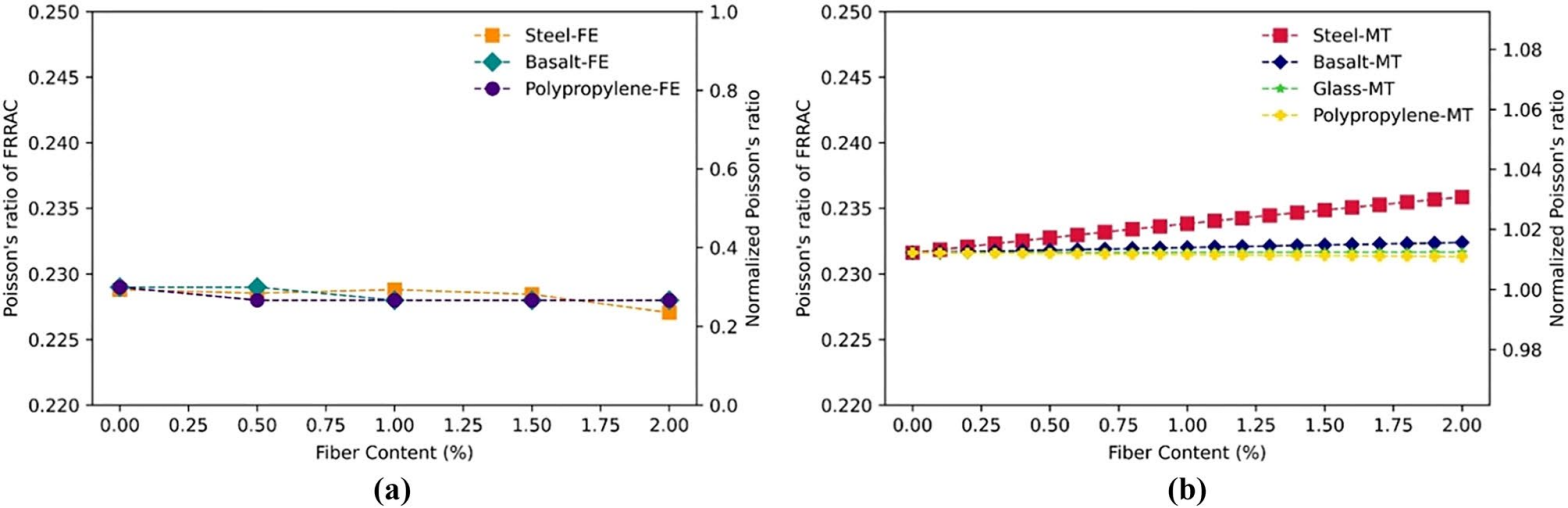
The Poisson’s ratio of FRRAC based on fiber content: (**a**) FE approach, (**b**) MT approach.

Increasing the fiber content in FRRAC typically results in an increase in its elastic modulus, given that high-modulus fibers including steel and basalt fibers are employed^55,61^. The reason for this can be attributed to the additional reinforcement and stiffness provided by these fibers. In contrast, lower-modulus fibers such as PP further reduce the elastic modulus of the FRRAC^64^. As seen in Fig. 9, the elastic modulus of FRRAC obtained by FE and MT approaches is increased by increasing steel, basalt, and glass fiber content. Accordingly, adding steel, basalt, and glass fibers to concrete elevates the elastic modulus of the FRRAC. This is because these fibers have a higher elastic modulus compared to the matrix, which makes the composite material stiffer and stronger, leading to an enhancement in the elastic modulus of the concrete. Such increase is higher for SFRRAC compared to BFRRAC, given that the elastic modulus of steel fibers is greater than that of the basalt fibers. On the contrary, the Young’s modulus of FRRAC is decreased as PP content is increased at both FE and MT approaches. This is associated with the fact that the elastic modulus of PFRRAC declines as PP fibers with lower modulus are added. Based on the results, both MT and FE approaches have estimated the variation trend of FRRAC elastic modulus in good accord. In addition, variation of the FRRAC Poisson’s ratio is expressed in Fig. 10. It is observed from Fig. 10 that Poisson’s ratio of SFRRAC in the MT approach surges with increasing fiber content, while that of the other fiber types remains almost unchanged. This is mainly because stiff steel fibers tend to confine the matrix transverse expansion under loading. Thus, concrete incurs higher transverse contraction strains with regard to longitudinal extension strains. Such mechanism obviously leads to an escalation in the Poisson's ratio. Nevertheless, the restricting influence of the fiber is barely experienced in the case of other fiber types. In this regard, there’s an increase of 5.5 and 2% in the elastic modulus in the FE method when steel and basalt fibers are added, respectively. However, the elastic modulus decreases by 2% in the case of PFRRAC. Likewise, in MT approach, the highest increase of 3% in the elastic modulus is experienced for SFRRAC. Besides, since glass fibers have almost similar elastic characteristics as to basalt fibers, the elastic modulus of both fiber types increases by 2%. The Poisson’s ratio of SFRRAC in the MT approach increases by 1.5% since the elastic property variations of all contributing phases are influential in this method. However, the Poisson’s ratio of the other fibers remains constant in both FE and MT methods.

### The effect of fiber elastic modulus

**Figure 11:**
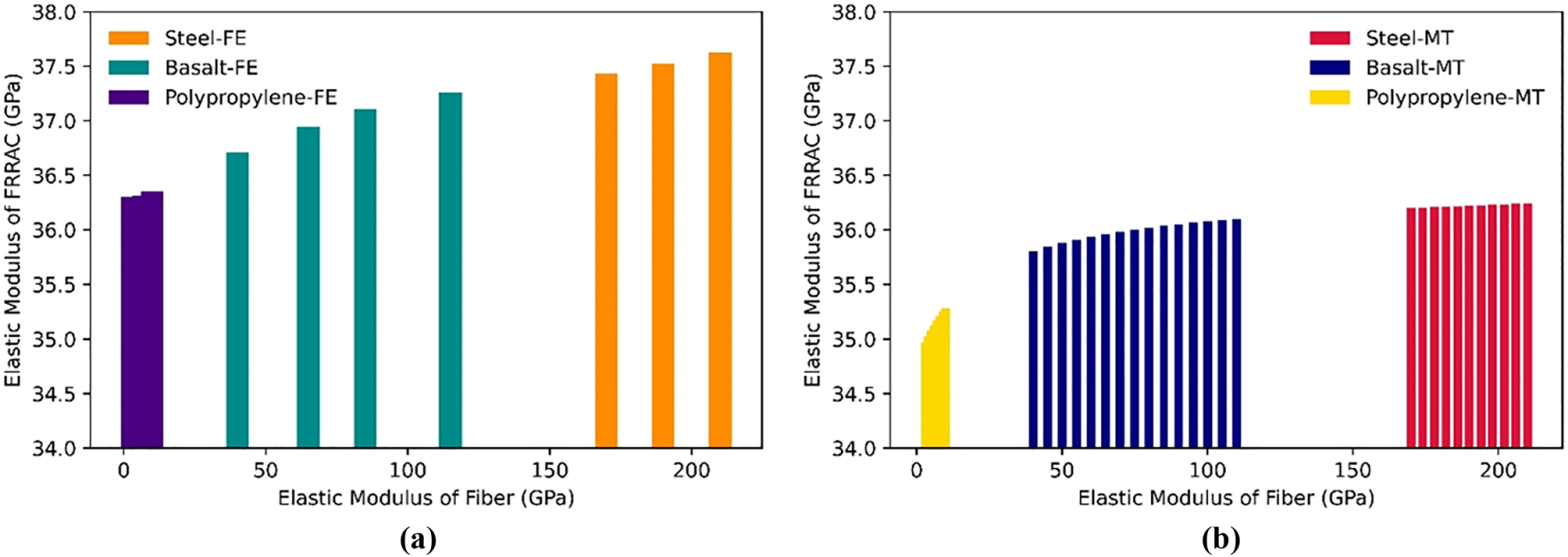
The elastic modulus of FRRAC based on fiber elastic modulus: (**a**) FE approach, (**b**) MT approach.

**Figure 12:**
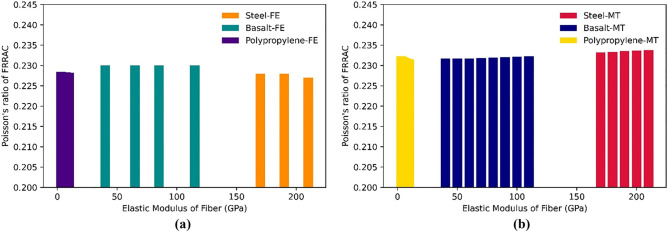
The Poisson’s ratio of FRRAC based on fiber elastic modulus: (**a**) FE approach, (**b**) MT approach.

The addition of fibers to FRRAC can give rise to substantial variations in its mechanical properties. In this respect, the type and volume fraction of the fibers implemented substantially impact the concrete elastic modulus. As for the fiber type, the commonly-used steel fibers have higher strength and stiffness compared to the more recently-developed basalt, glass, and PP fibers. The effect of the elastic modulus of different fiber types on the elastic modulus of FRRAC is demonstrated in Fig. 11. Accordingly, the elastic modulus of FRRAC rises as the elastic modulus of different fiber types increases in both MT and FE approaches. Although the elastic modulus of PFRRAC is lower than that of the other fiber types, it is seen that the elastic modulus of FRRAC is highly susceptible to an increase in the elastic modulus of PP fiber among other fiber types. PP fibers show relatively lower modulus of elasticity compared to steel and basalt. This connotes that PP fibers exhibit higher deformability exposed to loading before they begin to stiffen and resist deformation, thus, reducing the overall stiffness of the FRRAC. In like manner, the Poisson’s ratio of FRRAC remains almost unchanged for all fiber types, as depicted in Fig. 12. However, since the MT framework is affected by the elastic properties of all constituting phases, the increase on the Poisson's ratio of SFRRAC is more significant, given that the elastic modulus of steel fibers is much higher than the other fiber types. In the case of BFRRAC, the addition of the fibers with different elastic moduli may increase the stiffness and strength of the FRRAC, but it may not significantly affect its lateral deformation behavior. As a result, the Poisson's ratio of the composite remains relatively constant with the addition of the fibers, provided that the volume fraction and orientation of the fibers are consistent. The variations in the Poisson's ratio of GFRRAC in MT approach is similar to that of the BFRRAC, since the elastic properties of basalt fiber is almost similar to those of glass fiber. It should, however, be noted that the explicit influence of the fiber elastic modulus on the Poisson’s ratio relies not only on the fiber type, but also on the size, geometry, and orientation. In this respect, more specific investigation needs to be obtained through further testing and analyses.

### The effect of RA content

**Figure 13:**
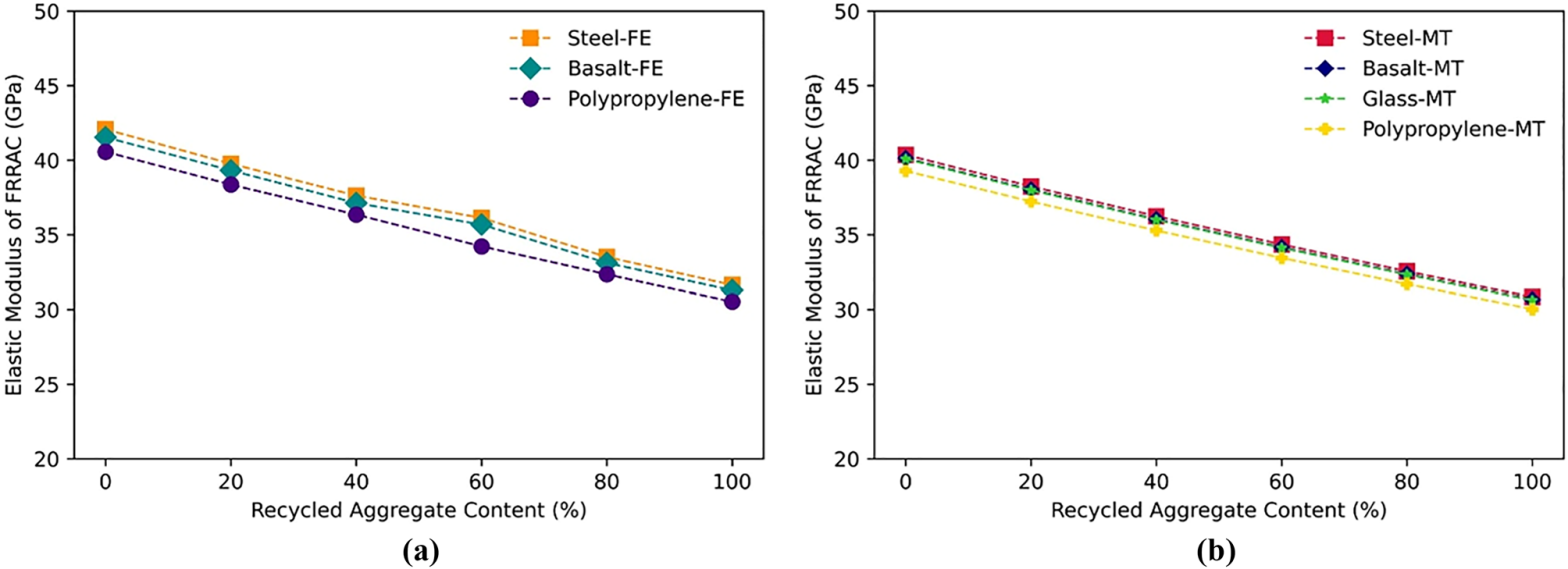
The elastic modulus of FRRAC based on RA content: (**a**) FE approach, (**b**) MT approach.

**Figure 14:**
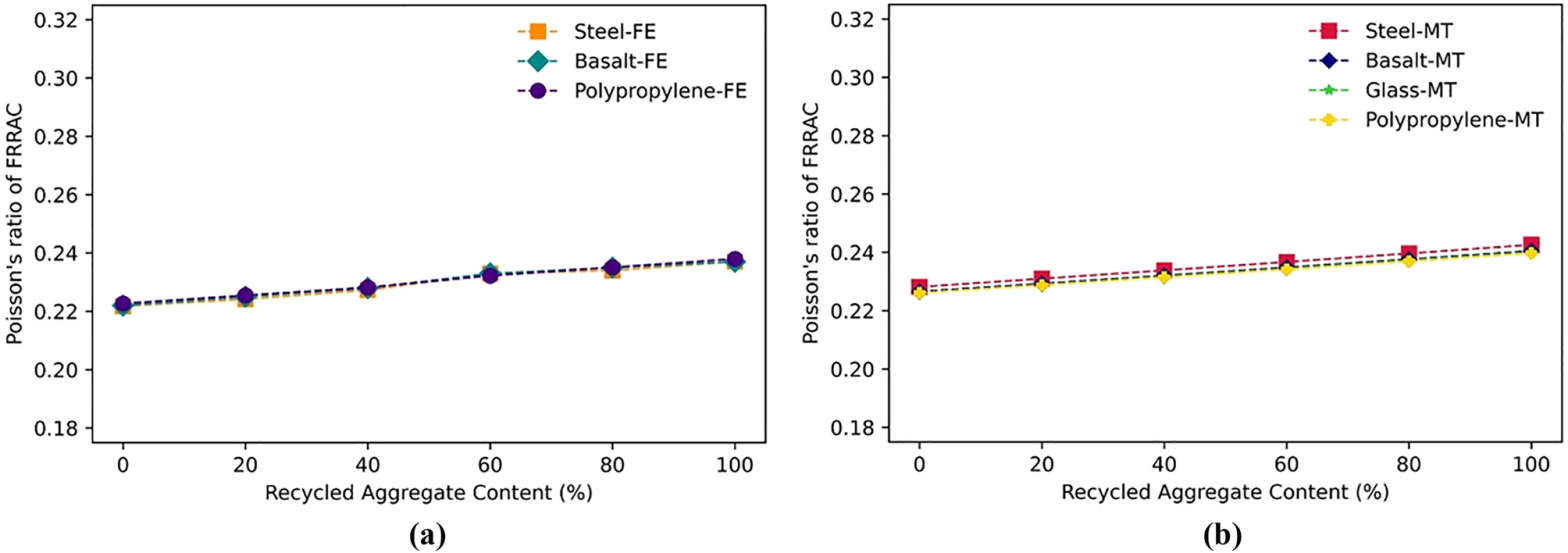
The Poisson’s ratio of FRRAC based on RA content: (**a**) FE approach, (**b**) MT approach.

As the RA content in concrete increases, the elastic modulus of the FRRAC typically decreases. This is attributed with the fact that RA features lower stiffness and strength capacity compared to NA, leading to reduced stiffness of the resulting composite. Accordingly, the reduction in the elastic modulus can be significant when higher RA contents are utilized in FRRAC. Figure 13 shows the declining trend of FRRAC elastic modulus with increasing RA content in both FE and MT approaches. This trend is in agreement with the previous experiments reported in the literature for steel, basalt, and PP fibers^55,61,64^. In this respect, the elastic modulus of concrete containing steel fibers is reduced by 25% in the FE approach as NA is totally replaced with RA. The corresponding reduction is approximately 24% in the MT homogenization approach. As such, different fiber types show similar declining trends. Moreover, the Poisson's ratio is increased constantly as the content of RA increases from 0 to 100% for all fiber types. A probable justification for an escalation in the Poisson's ratio of FRRAC with increasing RA content is the reduction in the elastic modulus of the FRRAC. RA generally has lower stiffness than NA owing to the adhered old and much porous mortar, resulting in a more porous and weaker composite. As the RA content in FRRAC increases, the overall stiffness is reduced, giving rise to a higher Poisson's ratio. The increasing trend of FRRAC Poisson’s ratio can be seen in Fig. 14 for all fiber types. In this regard, the Poisson’s ratio of SFRRAC increases by 8 and 7% in both FE and MT approaches, respectively.

### The effect of w/c ratio

**Figure 15:**
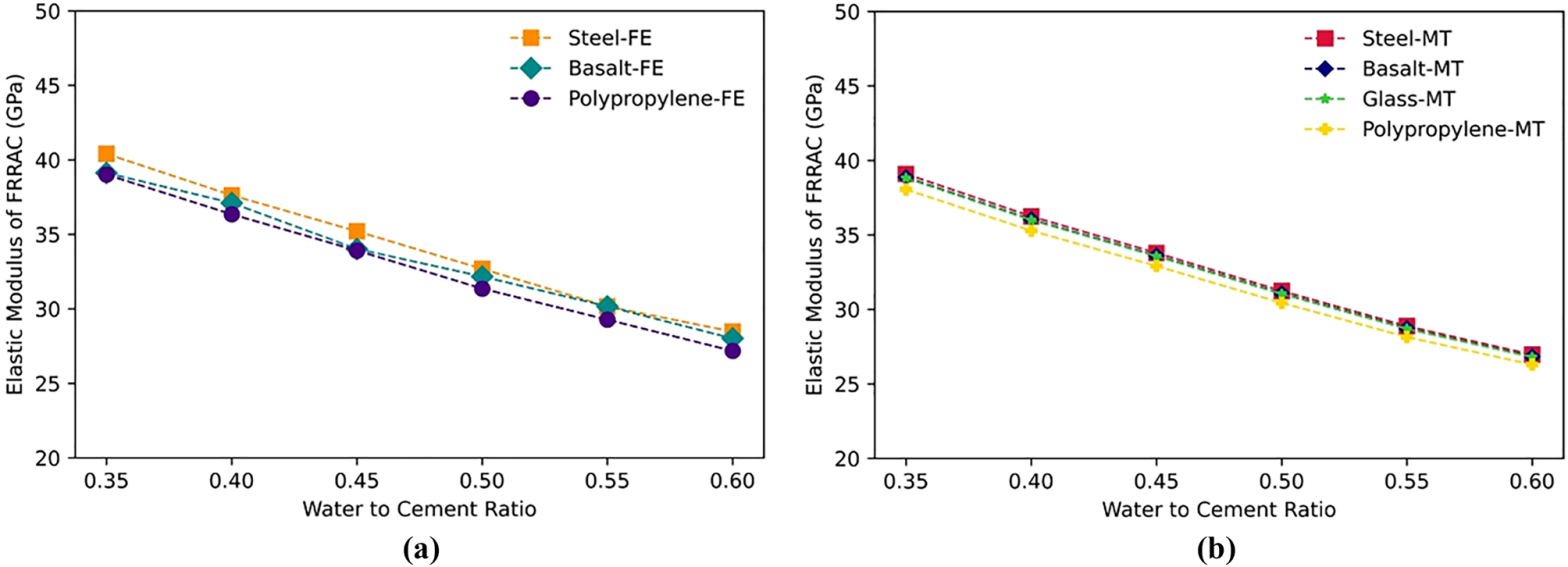
The elastic modulus of FRRAC based on w/c ratio: (**a**) FE approach, (**b**) MT approach.

**Figure 16:**
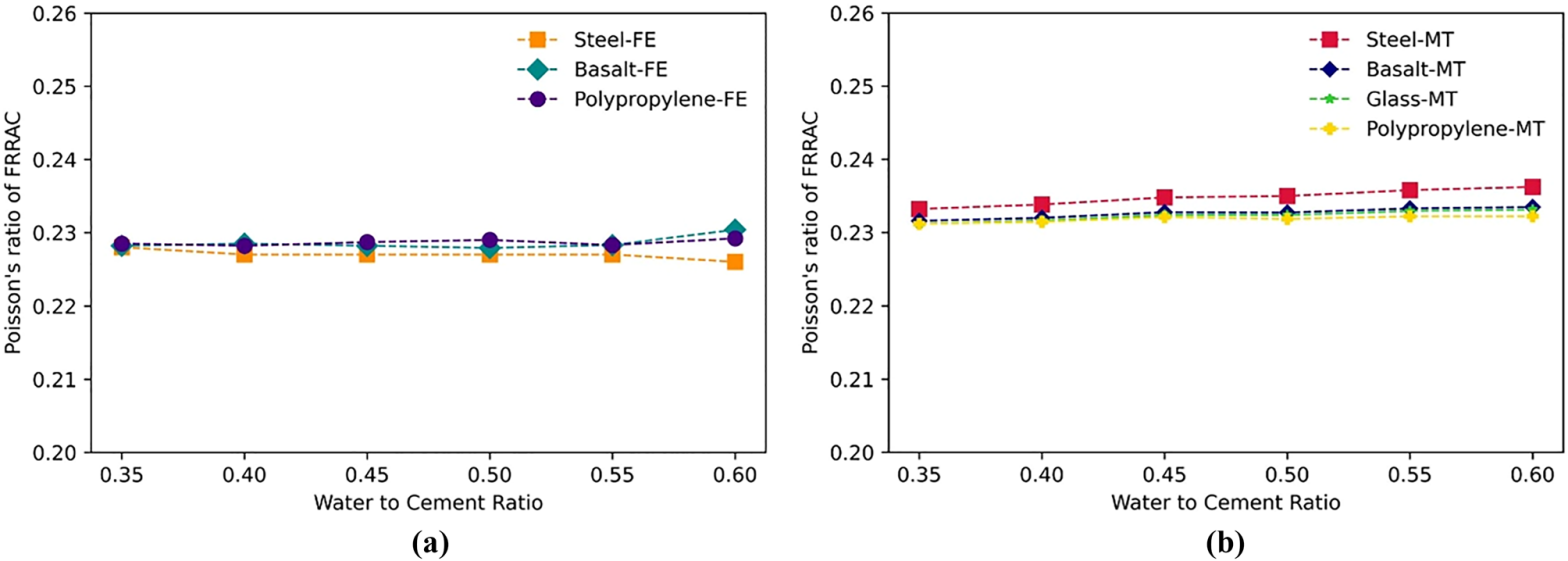
The Poisson’s ratio of FRRAC based on w/c ratio: (**a**) FE approach, (**b**) MT approach.

It is worth noting that the reduction range in the elastic modulus relies on peer factors other than the RA or fiber type and content, including the w/c. On this wise, increasing w/c in FRRAC results in a decline in the mechanical properties. The effect of the w/c on the elastic properties of FRRAC is given in Figs. 15 and 16. The elastic modulus of concrete experiences a sharp decrease with increasing w/c for all fiber types in both FE and MT approaches. The elastic modulus percentage of decrease in the FE method is 30, 29, and 30%, respectively for steel, basalt, and PP fibers as the w/c increases from 0.35 to 0.6. This reduction rate in the elastic modulus of FRRAC is 31% for all fiber types in the MT framework. Given that multiscale modeling approach was adopted in this study, w/c affects markedly the cement paste properties. Since cement paste properties are directly used in the upper scale to model the meso-scale composite material, the increase in w/c reduced the elastic modulus of the cement paste, and in turn, the FRRAC. This decreasing trend in the elastic modulus is also attributed to the effect of excess water in increasing the porosity of the concrete matrix regardless of the fiber type. This is mainly because a higher w/c entails more water content in the mixture, a lower composite strength, and increased matrix porosity. This brings about a reduction in the stiffness of the FRRAC, and in turn, a decrease in the elastic modulus. Furthermore, the w/c affects the fiber-matrix bond strength. Provided that the w/c is too high, the fibers may not be properly embedded in the matrix, and the fiber-matrix bond strength will be compromised. This leads to a limited fiber efficacy within the FRRAC, thus further reducing the elastic modulus. This is why the entire elastic modulus values are reduced with a similar slope for various fibers. In contrast, the Poisson’s ratio of FRRAC containing different fiber types in the MT approach gains measure as the w/c increases. As such, the Poisson’s ratio of the FRRAC with all fiber types increases by approximately 1% in both FE and MT methods with increasing the w/c from 0.35 to 0.6.

## Concluding remarks

The elastic properties of FRRAC were examined in this research using multi-scale analytic and numerical modeling approaches. Several models were created to evaluate the influence of variation in the Young’s modulus of each constituting FRRAC phases with the aid of the MT algorithm developed in Python programming language and the FE modeling approach. The effects of variations in fiber content, fiber elastic modulus, RA content, and w/c on the elastic modulus and Poisson’s ratio of each scale were also explored. Throughout the FE approach, mortar was modeled on a mesoscale. The results obtained on this scale were then used as input data on a macroscale. The predictions were validated using experimental data sets from previous studies. In this theory, the constituent phases were regarded spherical, with fiber as cylindrical, to capture the effect of elastic properties more accurately. Thus, spherical Eshelby tensor was implemented, while its algorithm was developed in Python. Likewise, in FEM approach, RVE models of FRRAC containing all phases were created in different sizes and mesh scales. Then, the effects of w/c ratio, fiber content, RA content, fiber elastic modulus, and sand volume fraction on the elastic properties of mortar (mesoscale) and FRRAC (macroscale) were examined. The results obtained in this research pave the way for an accurate understanding of the effect of FRRAC phases on mortar and concrete itself. They also propose an alternative to deplete the use of natural resources, recycle construction and demolition wastes, conserve the environment and enhance the economic sectors of concrete manufacturing. According to the 3D multi-scale modeling analysis conducted, the following results were drawn:

- The elastic modulus of FRRAC obtained by the FE and MT approaches is increased by increasing steel and basalt fiber content. This is because these fibers have a higher elastic modulus compared to the matrix, which makes the composite material stiffer and stronger, leading to an enhancement in the elastic modulus of the concrete.
- The Poisson’s ratio of SFRRAC in the MT approach surges with increasing fiber content, while that of the other fiber types remains almost unchanged. This is mainly because stiff steel fibers tend to confine the matrix transverse expansion under loading. Thus, concrete incurs higher transverse contraction strains with regard to longitudinal extension strains. Such mechanism obviously leads to an escalation in the Poisson's ratio. Nevertheless, the restricting influence of the fiber is barely experienced in the case of other fiber types.
- Although the elastic modulus of PFRRAC is lower than that of the other fiber types, it is seen that the elastic modulus of FRRAC is highly susceptible to an increase in the elastic modulus of PP fiber among other fiber types. PP fibers show relatively lower modulus of elasticity compared to steel and basalt. This connotes that PP fibers exhibit higher deformability exposed to loading before they begin to stiffen and resist deformation, thus, reducing the overall stiffness of the FRRAC.
- The elastic modulus of concrete containing steel fibers is reduced by 25% in the FE approach as NA are totally replaced with RA. The main reason is attributed to the fact that the existing old mortar adhered to RA significantly degrade the elastic modulus of the RAC. Furthermore, the Poisson’s ratio of SFRRAC increases by 8 and 7% in both FE and MT approaches, respectively. This is mainly because stiff steel fibers tend to confine the matrix transverse expansion under loading. Thus, concrete incurs higher transverse contraction strains with regard to longitudinal extension strains. Such mechanism obviously leads to an escalation in the Poisson's ratio.
- The Poisson’s ratio of PFRRAC decreases markedly when the elastic modulus of fiber is increased. This is mainly because of the reduction in the ratio of transverse strains to axial ones when PP and steel fibers are incorporated into the concrete mixture design.
- The elastic modulus of concrete experiences a sharp decrease with increasing w/c ratio for all fiber types in both FE and MT approaches. This is attributed to the effect of excess water in increasing the porosity of the concrete matrix regardless of the fiber type. This is why the entire elastic modulus values are reduced with a similar slope for various fibers.

## Data Availability

All data, models, and codes generated or used during the study appear in the submitted article.
